# Cotidianos, discursos e práticas em saúde: eugenia ontem e hoje

**DOI:** 10.1590/0102-311XPT191025

**Published:** 2026-07-24

**Authors:** Matheus Oliveira de Paula

**Affiliations:** 1 Fundação Oswaldo Cruz, Fiocruz, Rio de Janeiro, Brasil.

Muito presente no discurso político, pouco problematizada em seus enunciados, a eugenia não saiu do debate público, mas está nele enraizada em práticas sociais. A atualidade desta obra reside na distinção de um discurso tão comum que muitas vezes não percebemos o seu conteúdo. Para a crítica dessas práticas, o livro *Eugenia Ontem e Hoje* ^1^ contribui em três dimensões interligadas: (1) para a aproximação com a história do desenvolvimento da eugenia como campo de saber; (2) com a consequente crítica à eugenia e às práticas eugênicas; (3) na observância da permanência da eugenia no contexto contemporâneo.

Há algum tempo emprego a palavra eugenia, vinculada a determinadas dinâmicas higienistas que se reatualizam no cotidiano e nas práticas em saúde. Assim que conheci o livro resolvi adquirir. A leitura foi fácil, menos pela rigor sócio-histórico e densidade analítica, mais pela linguagem fluída e acessível. Os textos da coleção *Temas em Saúde*, Editora Fiocruz, têm esta proposta: acessibilidade e consistência teórico-metodológica. Em síntese, compreendi durante a leitura a extrema necessidade da discussão em função do enraizamento do discurso eugênico na contemporaneidade.

A afirmação do lugar das ciências sociais e humanas na saúde não refrata a permanência e a renovação de práticas eugênicas. Por isso, a obra visibiliza um debate que precisa estar no centro da agenda da saúde coletiva. A eugenia não foi abolida com o final da segunda guerra mundial, mas é reatualizada e permanece em práticas e enunciados políticos. No presente, como efeito do aprofundamento do neoliberalismo, as desigualdades permanecem constantes às das décadas passadas, por vezes são ampliadas e se revelam cada vez mais complexas. A liberdade de mercado como pressuposto às relações sociais amplia a privatização e individualização da saúde. Esse caldo cultural é favorável ao desenvolvimento de discursos conservadores, separatistas e eugênicos. O enfrentamento das desigualdades em saúde necessita de abordagens interseccionais, além da necessidade premente de observar e aprender com a história, com as continuidades.

Com base em um olhar sócio-histórico, Wegner resgata as bases da constituição da modernidade ocidental atreladas à eugenia. O colonialismo, o racismo científico e o nacionalismo, não por acaso, são o substrato da formação de um discurso eugênico. Ainda que durante a história alguns deslocamentos modificaram as práticas eugênicas, o cerne da suposta divisão biológica para processos sociais, culturais e políticos atravessa e constitui a formação discursiva eugênica.

O desenvolvimento da obra segue o resgate histórico da constituição da eugenia como um campo de saber. Originada como uma prática científica pautada na suposição de diferenças raciais, estruturada na arquitetura do racismo científico, a eugenia passa por um deslocamento que amplia a sua intervenção. Ou seja, o “outro” da prática eugênica, tido como “outras raças”, passa a observar dentro das “próprias raças” quem é o suposto “melhor” biologicamente. Assim, o “melhoramento da raça” é um fim em si mesmo dentro de todas as sociedades para a retórica eugênica. Essa descontinuidade enfatizada pelo autor marca a ciência eugênica na busca do “melhoramento” das populações nacionais.

A eugenia como ciência foi uma prática transnacional. No bojo do desenvolvimento científico, a retórica da modernidade e do desenvolvimento das nações foi estudada em termos biológicos, convergindo, negativamente, aspectos culturais e políticos com a dinâmica biológica. A resposta às questões emergentes, como a imigração no início do século XX, foram alvos de políticas eugênicas voltadas à esterilização, em alguns casos, e à escolha de determinados imigrantes, em outros.

A relação entre a eugenia e o nazismo é apresentada como um dos momentos em que as práticas eugênicas são esgarçadas, radicalizando-se, no nível de aniquilação de pessoas e populações. O autor não hierarquiza as práticas eugênicas, mas também não diminui o genocídio nazista. Efetivamente, ele mostra que o discurso eugenista é um continuum, devendo ser compreendido, criticado e rechaçado como um todo. A radicalização das práticas eugênicas foi constituída em determinado momento histórico e partiu de um discurso supostamente menos radical. Logo, recusar práticas que buscam o suposto “melhoramento da raça”, baseando-se em discursos biológicos, deve ser um horizonte contra todo e qualquer discurso eugênico na saúde pública.

No Brasil, a eugenia tem seus pontos de contato com o sanitarismo e o higienismo, relacionados pelo autor em dois capítulos do livro. Com uma descrição e interpretação histórico-sociológica das relações sociais e das questões político-econômicas, enfatiza os aspectos da eugenia relacionados à saúde pública e à instituição de políticas e práticas. Ainda, o mito da democracia racial forjou uma interpretação sociológica que foi instrumento da perpetuação das desigualdades raciais.

Foucault ^2^ é uma referência indispensável para compreender a instituição das práticas da medicina moderna, com base no conceito de biopolítica. O controle dos corpos atinge graus cada vez mais complexos dentro da sociedade contemporânea. O diálogo com conceitos foucaultianos é explorado de maneira oportuna, mobilizando ainda a interlocução com autores contemporâneos que atualizam a biopolítica, como Mbembe ^3^ empregando o conceito de necropolítica. Para a problematização da eugenia, relacionar a gestão da vida, os mecanismos e estratégias de poder, com definição dos corpos que podem morrer, complexifica a análise das estratégias do exercício do poder contemporâneo e das formas de expressão da eugenia em discursos.

As políticas sociais (educação, assistência social, previdência), de modo geral, na sua interlocução com a política de saúde poderiam ter merecido mais espaço na obra. Já que a reprodução de práticas eugênicas é constituída em diferentes espaços sociais e instituições, historicamente no Brasil a política de saúde teve uma relação muito próxima com a política de educação (sobretudo com o discurso higienista). Todavia, a aproximação entre eugenia e Estado de Bem-Estar Social (*Welfare State*) - nos países escandinavos - interpela os valores de democracia e igualdade construídos sobre esses países e suas políticas, pois mostra como a constituição do sistema de proteção social deles foi baseado em práticas eugênicas.

Não superamos a estrutura racista que constitui a sociedade brasileira. Ainda, é significativa a presença de um determinismo biológico nos discursos cotidianos. No campo da saúde coletiva brasileira, os acúmulos realizados durantes o final do século XX e durante o século XXI, baseando-se na teoria crítica e na perspectiva decolonial, enfatizam a construção social, histórica e cultural das relações sociais, portanto, as dimensões sociais do adoecimento com base em categorias como a determinação social da saúde e, mais recentemente, a interseccionalidade. Essas abordagens enfatizam a dinâmica de produção social das desigualdades em saúde.

As propostas reprodutoras do ideal eugênico estão presentes na política brasileira. Em anos recentes, o chefe do Poder Executivo de um município defendeu que “*tem que começar a castrar essas meninas. Controlar essa população*” ^4^. A proposta é endereçada a um determinado público de mulheres, as quais o Estado, supostamente, deve interferir mais sistematicamente em seus corpos. Ainda que a globalidade do patriarcado afete as mulheres no geral, é no específico, em algumas identidades, que observamos a reprodução de violências interseccionais, em mulheres pobres e negras. Não é estranho que a indignação gerada e a visibilidade pública sobre a fala não tenham levado à cassação do mandato, à interdição desse discurso. Sim, interdição. Na busca por sociedades democráticas que valorizem a diversidade e a pluralidade, um discurso que encerra o outro não pode ser permitido.

As expressões de práticas eugênicas no presente são reatualizadas com o desenvolvimento científico e tecnológico. As tecnologias de reprodução assistida, os estudos genômicos e os testes genéticos são áreas de necessária vigilância ética e epistemológica das possibilidades e impactos nas populações humanas. No contemporâneo, sob a égide neoliberal, as práticas de saúde aprofundam uma intervenção voltada ao controle dos corpos, individualização do cuidado e medicalização da vida. Por isso, na saúde coletiva, campo de saberes e práticas, é necessário que radicalizemos a abordagem e as interpretações que rechacem a reprodução de discursos e práticas eugênicas. Com as expressões contemporâneas dos discursos de “melhoramento racial”, o autor encerra os debates da obra, e nos deixa um horizonte aberto para observar as expressões eugênicas no cotidiano. Mais do que isso, no campo da saúde coletiva, nos oferece recursos para a crítica da eugenia ontem e hoje.
